# Depletion of Psoas Muscle Mass after Systemic Chemotherapy Is Associated with Poor Prognosis in Patients with Unresectable Pancreatic Cancer

**DOI:** 10.3390/cancers13153860

**Published:** 2021-07-31

**Authors:** Naoto Iwai, Takashi Okuda, Kohei Oka, Junichi Sakagami, Taishi Harada, Tomoya Ohara, Chie Hattori, Masashi Taniguchi, Hiroaki Sakai, Tasuku Hara, Toshifumi Tsuji, Toshiyuki Komaki, Keizo Kagawa, Osamu Dohi, Hiroaki Yasuda, Yoshito Itoh

**Affiliations:** 1 Department of Gastroenterology and Hepatology, Fukuchiyama City Hospital, Fukuchiyama 620-8505, Japan; okudatti@koto.kpu-m.ac.jp (T.O.); kooka@koto.kpu-m.ac.jp (K.O.); junichi@koto.kpu-m.ac.jp (J.S.); chattori@koto.kpu-m.ac.jp (C.H.); masa1223@koto.kpu-m.ac.jp (M.T.); sakaihiroaki6830@gmail.com (H.S.); t-hara@koto.kpu-m.ac.jp (T.H.); tsuji626@koto.kpu-m.ac.jp (T.T.); t171koma@gmail.com (T.K.); k-kag@fukuchiyama-hosp.jp (K.K.); 2 Department of Molecular Gastroenterology and Hepatology, Graduate School of Medical Science, Kyoto Prefectural University of Medicine, Kyoto 602-8566, Japan; tohara@koto.kpu-m.ac.jp (T.O.); osamu-d@koto.kpu-m.ac.jp (O.D.); hiyasuda@koto.kpu-m.ac.jp (H.Y.); yitoh@koto.kpu-m.ac.jp (Y.I.); 3 Department of Medical Oncology, Fukuchiyama City Hospital, Fukuchiyama 620-8505, Japan; tharada5@hotmail.co.jp

**Keywords:** psoas muscle mass index, chemotherapy, unresectable pancreatic cancer, prognosis

## Abstract

**Simple Summary:**

The psoas muscle mass index (PMI) is a simplified tool used in the quantification of skeletal muscle. The aim of our study was to investigate whether the PMI at diagnosis or its decrease during chemotherapy can influence the prognosis of unresectable pancreatic cancer. The median overall survival (OS) was 278.0 (95% confidence interval (CI), 199.1–356.9) days in the high-PMI group, and 221.0 (95% CI, 90.9–351.1) days in the low-PMI group (*p* = 0.329). The median OS was 347.0 (95% CI, 289.1–404.9) days in the group without PMI decrease and 172.0 (95% CI, 129.8–214.2) days in the group with PMI decrease (*p* = 0.001). We determined that the PMI at diagnosis was not associated with OS in patients with unresectable pancreatic cancer receiving systemic chemotherapy, whereas PMI decrease during chemotherapy influenced the OS.

**Abstract:**

The impact of the psoas muscle mass index (PMI) on survival is still poorly understood in unresectable pancreatic cancer. Thus, we aimed to investigate whether the PMI at diagnosis or its decrease during chemotherapy can influence the prognosis of unresectable pancreatic cancer. The data of 100 patients were analyzed, and they were divided into two groups according to the median PMI in each sex. Subsequently, 72 patients undergoing computed tomography (CT) within 30–100 days from CT at diagnosis were evaluated in terms of PMI change rate, and divided into two groups based on the median. We evaluated the clinical characteristics and outcomes in terms of the PMI at diagnosis or its decrease during chemotherapy. The median PMI was 5.00 in males, and 3.66 in females. The median overall survival (OS) was 278.0 days in the high-PMI group and 221.0 days in the low-PMI group (*p* = 0.329). The median PMI change rate was −2.4%. The median OS was 347.0 days in the group without PMI decrease and 172.0 days in the group with PMI decrease (*p* = 0.001). We determined that a pivotal prognostic factor was not the PMI at diagnosis, but rather PMI decrease during chemotherapy in unresectable pancreatic cancer.

## 1. Introduction

Pancreatic cancer is one of the most aggressive malignancies, and it has the lowest survival rate compared to other cancer types [1]. There are no established screening methods for early detection of pancreatic cancer [2]. Thus, the clinical stage could easily reach an unresectable stage. Despite the advances in chemotherapy, the median overall survival (OS) is less than one year in patients with metastatic pancreatic cancer [3,4,5].

Sarcopenia was originally defined as an age-related decline in skeletal mass, whereas secondary sarcopenia was characterized by loss of skeletal mass due to conditions such as inflammatory diseases and malignant diseases [6]. Computed tomography (CT) images are used to evaluate the skeletal mass or body composition of patients with cancer [7], and the CT image analysis can contribute to identifying the association between sarcopenia and pancreatic cancer [8]. However, many previous studies have focused on patients with pancreatic cancer undergoing surgical resection [9,10,11,12]. The preoperative psoas muscle mass index (PMI) is reported to be a pivotal prognostic factor in resectable pancreatic cancer [10]. The PMI is a simplified and quick tool for quantifying skeletal muscle using the manual tracing technique. Regarding unresectable pancreatic cancer, sarcopenia at diagnosis or depletion of skeletal muscle during chemotherapy was recently identified as a risk factor for poor OS [13,14,15,16,17]. The skeletal muscle index (SMI) is used to evaluate the body composition of patients with pancreatic cancer receiving chemotherapy; however, the impact of the PMI on the survival of patients with unresectable pancreatic cancer remains unclear [17].

Therefore, in the present study, we aimed to investigate whether the PMI at diagnosis or its decrease during chemotherapy can influence the prognosis of patients with unresectable pancreatic cancer.

## 2. Materials and Methods

**Figure 1:**
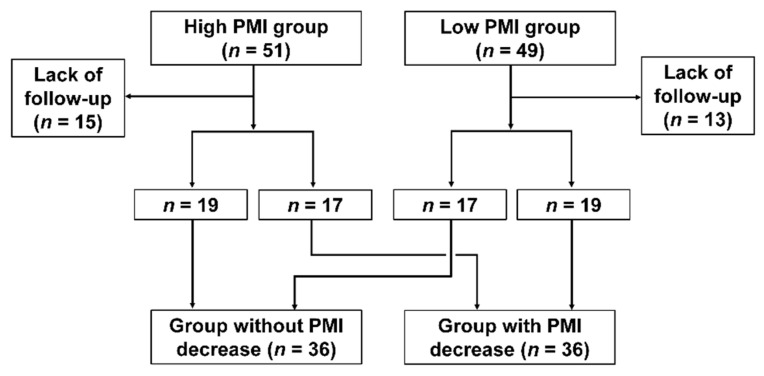
Flow diagram.

We retrospectively registered 218 patients with pancreatic cancer who were admitted to Fukuchiyama City Hospital between June 2006 and September 2020. Of the enrolled patients, 65 who received the best supportive care, 34 who underwent surgery, and 19 who received chemoradiotherapy or radiotherapy were excluded. Therefore, 100 patients (23 with locally advanced pancreatic cancer and 77 with metastatic pancreatic cancer) who received systemic chemotherapy were included. We divided the patients into two groups according to the median PMI values in each sex, and evaluated the clinical characteristics between the two groups: the high-PMI group (51 patients) and the low-PMI group (49 patients). Additionally, based on a previous study, the PMI change rate was evaluated in 72 patients undergoing CT within 30–100 days of the CT at diagnosis [18]. We divided those patients into two groups according to the median PMI change rate, and assessed the clinical characteristics between the two groups: patients without PMI decrease (36 patients) and those with PMI decrease (36 patients) (Figure 1).

We evaluated baseline characteristics, such as age, sex, Eastern Cooperative Oncology Group Performance Status (ECOG-PS), and body mass index (BMI), in addition to carbohydrate antigen 19-9 (CA19-9) levels, the platelet-to-lymphocyte ratio (PLR) [19], the neutrophil-to-lymphocyte ratio (NLR) [20], the C-reactive protein-to-albumin ratio (CRP/Alb) [21], and the prognostic nutritional index (PNI) [22], at the time of diagnosis. We also evaluated lesion characteristics regarding location, clinical stage, and the number of metastatic sites using the 7th edition of the General Rules for the Study of Pancreatic Cancer [23]. Regarding treatment, we assessed the first-line chemotherapy regimen. Regarding clinical outcomes, the treatment responses of the 72 patients who underwent CT within 30–100 days of the CT at diagnosis were evaluated using pre-treatment and the first CT images after the chemotherapy induction. Treatment response was classified as complete response, partial response, stable disease, and progressive disease (PD). When we assessed the clinical outcomes of the patients without or with PMI decrease, the change rates of CA19-9, PLR, NLR, CRP/Alb, and PNI were calculated using the values obtained at diagnosis and during the treatment response evaluation.

OS was calculated from the date of the CT scan at diagnosis to that of last follow-up or death, whereas progression-free survival (PFS) was calculated from the date of the CT scan at diagnosis to that of PD or death.

The PMI was retrospectively assessed using CT images. The cross-sectional areas at the third lumbar vertebrae level in the inferior direction were calculated using the manual tracing technique with SYNAPSE SCOPE (Fujifilm Medical, Tokyo, Japan), and the PMI value (cm^2^/m^2^) was normalized to individual height (meters). For the PMI change rate, the PMI values on the first CT images after chemotherapy induction were compared with the pre-treatment values.

Statistical analyses were performed using SPSS Statistics versions 26.0 and 27.0 (IBM Japan, Tokyo, Japan). A *p*-value < 0.05 was considered as statistically significant. Numerical data are presented as median and range. Comparisons between groups were analyzed using the Mann–Whitney U test and the chi-square test. The OS and PFS rates were estimated using Kaplan–Meier’s curves, and the log rank analyses were performed to ascertain the significance.

## 3. Results

### 3.1. Clinical Characteristics of the High- and Low-PMI Groups

Table 1 shows the clinical characteristics of the high- and low-PMI groups. The median PMI value was 5.00 (cm^2^/m^2^) in males, and 3.66 (cm^2^/m^2^) in females. Using the median PMI value, the high-PMI group was defined as the patients with a PMI value ≥5.00 (males) or ≥3.66 (females), whereas the low-PMI group was defined as those with a PMI value <5.00 (males) or <3.66 (females). There were no significant differences in age, sex, ECOG-PS, BMI, location, clinical stage, number of metastatic sites, CA19-9 levels, NLR, CRP/Alb, or PNI between the groups. The PLR in the low-PMI group was significantly higher than that in the high-PMI group (*p* = 0.044).

### 3.2. Comparison of the Clinical Outcomes between the High- and Low-PMI Groups

**Figure 2:**
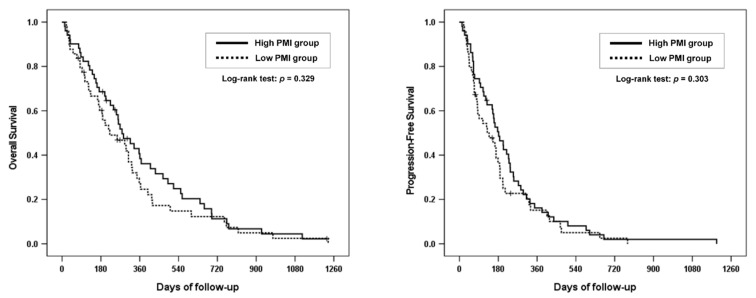
Overall survival and progression-free survival of the high- and low-PMI groups. PMI; psoas muscle mass index.

Table 2 shows the clinical outcomes of the high- and low-PMI groups. Regarding the first-line chemotherapy regimen, approximately 60% of the patients in the two groups received gemcitabine monotherapy. There were no differences in treatment regimen or response. The median follow-up period of the enrolled patients was 248.5 (range, 12–1234) days. During the follow-up period, 90 patients died. The median OS was 278.0 (95% confidence interval (CI), 199.1–356.9) days in the high-PMI group, and 221.0 (95% CI, 90.9–351.1) days in the low-PMI group. The six-month and one-year OS rates in the high-PMI group were 68.6% and 38.4%, respectively, whereas those in the low-PMI group were 60.2% and 24.6%, respectively (*p* = 0.329; Figure 2). There was no significant difference in OS between the groups. The median PFS was 182.0 (95% CI, 131.9–232.1) days in the high-PMI group and 129.0 (95% CI, 49.5–208.5) days in the low-PMI group. The six-month and one-year PFS rates in the high-PMI group were 50.6% and 16.2%, respectively, whereas those in the low-PMI group were 36.4% and 15.2%, respectively (*p* = 0.303; Figure 2). There was no significant difference in PFS between the groups.

### 3.3. Clinical Characteristics of the Patients with and without PMI Decrease

Table 3 shows the clinical characteristics of patients with and without PMI decrease. The median PMI change rate was −2.4% (range, −39.3 to 18.4%). Using the median PMI change rate, the group without PMI decrease was defined as the patients with a PMI change rate ≥−2.4%, whereas the group with PMI decrease was defined as those with a PMI change rate <−2.4%. The median period from the CT at diagnosis to the first CT after chemotherapy induction was 66.5 (range, 30–100) days. There were no significant differences in age, sex, ECOG-PS, BMI at diagnosis, location, clinical stage, or number of metastatic sites between the groups.

### 3.4. Clinical Outcomes according to the PMI Change Rate

Table 4 shows the clinical outcomes according to the PMI change rate. There were no significant differences in first-line chemotherapy regimen, CA19-9 change rate, PLR change rate, or NLR change rate. Regarding treatment response, the group without PMI decrease showed a better response than the group with PMI decrease (*p* < 0.001). Notably, only 2.8% of patients without PMI decrease had PD, whereas 63.9% of patients with PMI decrease had PD. The CRP/Alb change rate in the group without PMI decrease was significantly lower than that in the group with PMI decrease (*p* < 0.001). The PNI change rate in the group without PMI decrease was significantly higher than that in the group with PMI decrease (*p* = 0.022).

**Figure 3:**
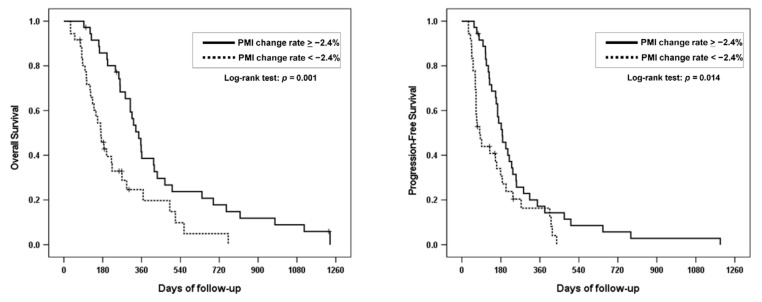
Overall survival and progression-free survival according to PMI change rate.

The median OS was 347.0 (95% CI, 289.1–404.9) days in the group without PMI decrease and 172.0 (95% CI, 129.8–214.2) days in the group with PMI decrease. The six-month and one-year OS rates in the group without PMI decrease were 85.8% and 38.6%, respectively, whereas those in the group with PMI decrease were 45.8% and 24.7%, respectively (*p* = 0.001; Figure 3). The median PFS was 187.0 (95% CI, 143.0–231.0) days in the group without PMI decrease and 82.0 (95% CI, 49.4–114.6) days in the group with PMI decrease. The six-month and one-year PFS rates in the group without PMI were 54.4% and 17.2%, respectively, whereas, those in the group with PMI decrease were 30.6% and 16.3%, respectively (*p* = 0.014; Figure 3).

## 4. Discussion

In the present study, we determined that the PMI at diagnosis was not associated with OS and PFS in patients with unresectable pancreatic cancer receiving systemic chemotherapy, whereas PMI decrease during chemotherapy influenced the OS and PFS. The patients without PMI decrease showed a better response than those with PMI decrease. However, PMI decrease itself was not related to ECOG-PS, BMI at diagnosis, clinical stage, or first-line chemotherapy regimen.

Previous reports have shown that the prevalence of skeletal muscle depletion could affect the prognosis in patients with solid tumor patients [24,25,26]. Furthermore, the association between sarcopenia and pancreatic cancer has been elucidated. Some previous studies revealed that sarcopenia could affect prognosis or complications after surgical resection [9,10,11,12]. Recently, the relationship between sarcopenia and treatment response or OS in unresectable or recurrent pancreatic cancer has been of growing interest [13,14,15,16,17,27]. Furthermore, the advances in chemotherapy regimens have resulted in a switch from gemcitabine monotherapy to intensive chemotherapy with agents such as FOLFIRINOX and gemcitabine plus nab-paclitaxel. However, the evaluation method or impact on prognosis of sarcopenia remains to be clarified in this novel chemotherapy era. We previously conducted a study that investigated inflammatory response and demonstrated that the NLR was an important prognostic factor for unresectable pancreatic cancer [28]. This study focused on the relationship between the PMI and prognosis of patients with unresectable pancreatic cancer.

In this study, there were no differences in age, clinical stage, or CA19-9 levels between the high- and low-PMI groups in accordance with previous studies [14,17]. Moreover, no significant difference was observed in BMI between the two groups, unlike the findings reported previously [13,17]. The prevalence of overweight or obese sarcopenia in the study populations may explain this discrepancy [8]. The prevalence of overweight or obese patients (BMI >23 kg/m^2^) in the low-PMI group was 24.5%, whereas that in the high-PMI group was 29.4%. In addition, the pivotal prognostic factor was not the PMI at diagnosis, but rather PMI decrease during chemotherapy in patients with unresectable pancreatic cancer. The IMPACT study also showed that early depletion of SMI was a negative prognostic marker in advanced pancreatic cancer, whereas sarcopenia at the time of diagnosis was not a negative prognostic marker [16]. Our findings may suggest that a low PMI at diagnosis should not be considered as a contraindication for chemotherapy in patients with unresectable pancreatic cancer. Inconsistent with our results, Ishii et al. showed that the PMI at diagnosis was associated with OS. In their study, most of the patients received gemcitabine or S-1 monotherapy, and BMI was significantly different between the low- and high-PMI groups. The differences in patients’ backgrounds or chemotherapy regimens could explain the discrepancies.

We used the PMI as the parameter of skeletal muscle because it can be simply calculated using the manual tracing technique. The PMI has been reported to be a useful prognostic predictor of pancreatic cancer [10,17,27]. Hamaguchi et. al. suggested that the cutoff values for the PMI were 6.36 cm^2^/m^2^ for males and 3.92 cm^2^/m^2^ for females among the healthy young adult Asian population [29]. However, little evidence is available regarding the ideal cutoff values in unresectable pancreatic cancer. Thus, the median values in males and females in this study were used as the cutoff values, as in a previous study [17].

PMI decrease during chemotherapy was not related to age, ECOG-PS, clinical stage, or first-line chemotherapy regimen. Furthermore, the CRP/Alb and PNI change rates were significantly associated with PMI decrease. These results suggest that the PMI may strongly reflect nutritional status. Furthermore, prevention of PMI decrease through nutritional support or rehabilitation may be a useful therapeutic strategy to improve the prognosis of patients with pancreatic cancer receiving chemotherapy. Anamorelin, a selective ghrelin receptor agonist, has been proven to be effective in improving lean body mass, body weight, and nutritional state in patients with advanced gastrointestinal cancer [30]. As our results showed, patients with unresectable pancreatic cancer frequently lose skeletal muscle because pancreatic cancer itself can lead to systemic inflammation, which results in muscle protein catabolism; furthermore, chemotherapy can induce anorexia. Thus, the PMI maintenance by anamorelin may be a promising therapeutic strategy for secondary sarcopenia in pancreatic cancer.

The present study has some limitations. First, this study was a single-center, retrospective study with a small sample size; thus, the possibility that an important factor might have been underestimated cannot be ruled out. In addition, the first CT timing after chemotherapy induction could not be determined because of the retrospective study design. Therefore, a large multicenter prospective study is required to confirm our results. Second, the optimal PMI cutoff value was not determined. Further investigation should be conducted to establish an optimal PMI cutoff value in patients with unresectable pancreatic cancer. Third, this study only included Japanese patients with unresectable pancreatic cancer; thus, our findings should be validated in other populations. Fourth, approximately 60% of the enrolled patients received gemcitabine monotherapy due to the study period; thus, our results should be also confirmed in patients receiving the current standard therapy (FOLFIRINOX and gemcitabine plus nab-paclitaxel).

## 5. Conclusions

This study determined that a pivotal prognostic factor was not the PMI at diagnosis, but rather PMI decrease during chemotherapy in unresectable pancreatic cancer. These findings suggest that a low PMI at diagnosis should not be considered as a contraindication for chemotherapy, and prevention of PMI decrease may be a useful therapeutic strategy to improve the prognosis of patients with unresectable pancreatic cancer.

## Figures and Tables

**Table 1:** Comparison of the clinical characteristics between the high- and low-PMI groups.

Characteristics	High-PMI Group		Low-PMI Group		*p*-Value
No. of patients, *n*	51		49		
Age (range)	71	(37–87)	72	(54–87)	0.238
Sex, *n* (%)					
Male	24	(47.1)	23	(46.9)	1.000
Female	27	(52.9)	26	(53.1)	
ECOG-PS, *n* (%)					
0	17	(33.3)	19	(38.8)	0.720
≥1	34	(66.7)	30	(61.2)	
BMI (range)	21.0	(13.3–30.3)	20.3	(15.8–28.6)	0.208
Location, *n* (%)					
Head	24	(47.1)	21	(42.9)	0.825
Body or tail	27	(52.9)	28	(57.1)	
Stage, *n* (%)					
III	15	(29.4)	8	(16.3)	0.188
IV	36	(70.6)	41	(83.7)	
No. of metastatic sites, *n* (%)					
0	15	(29.4)	8	(16.3)	0.068
1	17	(33.3)	16	(32.7)	
2	11	(21.6)	17	(34.7)	
3	4	(7.8)	8	(16.3)	
≥4	4	(7.8)	0	(0.0)	
CA19-9 (range)	3971.3	(1.0–600,000.0)	2269.8	(2.0–121,200.0)	0.650
PLR (range)	155.75	(41.78–344.92)	185.24	(62.32–585.55)	0.044
NLR (range)	3.50	(0.82–23.64)	4.34	(1.00–13.04)	0.203
CRP/Alb (range)	0.16	(0.00–8.81)	0.19	(0.01–4.80)	0.560
PNI (range)	46.31	(29.90–63.42)	43.66	(29.61–56.66)	0.281

PMI, psoas muscle mass index; ECOG, Eastern Cooperative Oncology Group; PS, performance status; BMI, body mass index; CA19-9, carbohydrate antigen 19-9; PLR, platelet-to-lymphocyte ratio; NLR, neutrophil-to-lymphocyte ratio; CRP, C-reactive protein; Alb, albumin; PNI, prognostic nutritional index. The continuous variables are expressed as medians and ranges, and the categorical variables are expressed as numbers and percentages.

**Table 2:** Comparison of the clinical outcomes between the high- and low-PMI groups.

Characteristics	High-PMI Group		Low-PMI Group		*p*-Value
No. of patients, *n*	51		49		
Treatment, *n* (%)					
Gemcitabine	31	(60.8)	29	(59.2)	0.284
modified FOLFIRINOX	10	(19.6)	13	(26.5)	
Gemcitabine + nab-PTX	5	(9.8)	5	(10.2)	
Gemcitabine + erlotinib	2	(3.9)	0	(0.0)	
Gemcitabine + S1	3	(5.9)	0	(0.0)	
S1 + oxaliplatin	0	(0.0)	1	(2.0)	
S1	0	(0.0)	1	(2.0)	
Response *, *n* (%)					
Complete response	0	(0.0)	0	(0.0)	0.514
Partial response	2	(3.9)	4	(8.2)	
Stable disease	24	(47.1)	18	(36.7)	
Progressive disease	10	(19.6)	14	(28.6)	
Unevaluable	15	(29.4)	13	(26.5)	

PMI, psoas muscle mass index; nab-PTX, nab-paclitaxel. The categorical variables are expressed as numbers and percentages. * Regarding response, 72 patients who underwent computed tomography (CT) within 30–100 days of the first CT at diagnosis were evaluated.

**Table 3:** Comparison of the clinical characteristics in the patients without or with PMI decrease.

Characteristics	Patients without PMI Decrease		Patients with PMI Decrease		*p*-Value
No. of patients, *n*	36		36		
Age (range)	72	(37–87)	72	(54–86)	0.335
Sex, *n* (%)					
Male	19	(52.8)	20	(55.6)	1.000
Female	17	(47.2)	16	(44.4)	
ECOG-PS, *n* (%)					
0	13	(36.1)	13	(36.1)	1.000
≥1	23	(63.9)	23	(63.9)	
BMI at diagnosis (range)	20.7	(15.7–30.3)	20.4	(13.3–28.6)	0.884
Location, *n* (%)					
Head	19	(52.8)	14	(38.9)	0.344
Body or tail	17	(47.2)	22	(61.1)	
Stage, *n* (%)					
III	10	(27.8)	8	(22.2)	0.785
IV	26	(72.2)	28	(77.8)	
No. of metastatic sites, *n* (%)					
0	10	(27.8)	8	(22.2)	0.616
1	13	(36.1)	10	(27.8)	
2	10	(27.8)	11	(30.6)	
3	2	(5.6)	6	(16.7)	
≥4	1	(2.8)	1	(2.8)	

PMI, psoas muscle mass index; ECOG, Eastern Cooperative Oncology Group; PS, performance status; BMI, body mass index. The continuous variables are expressed as medians and ranges, and the categorical variables are expressed as numbers and percentages.

**Table 4:** Comparison of the clinical outcomes of the patients without or with PMI decrease.

Characteristics	Patients without PMI Decrease		Patients with PMI Decrease		*p*-Value
No. of patients, *n*	36		36		
Treatment, *n* (%)					
Gemcitabine	18	(50.0)	23	(63.9)	0.574
modified FOLFIRINOX	10	(27.8)	8	(22.2)	
Gemcitabine + nab-PTX	6	(16.7)	3	(8.3)	
Gemcitabine + erlotinib	1	(2.8)	0	(0.0)	
Gemcitabine +S1	1	(2.8)	1	(2.8)	
S1 + oxaliplatin	0	(0.0)	1	(2.8)	
Response, *n* (%)					
Complete response	0	(0.0)	0	(0.0)	<0.001
Partial response	5	(13.9)	1	(2.8)	
Stable disease	30	(83.3)	12	(33.3)	
Progressive disease	1	(2.8)	23	(63.9)	
CA19-9 change rate (range)	0.63	(0.04–2.05)	0.92	(0.00–7.36)	0.191
PLR change rate (range)	0.90	(0.35–10.46)	0.95	(0.24–4.50)	0.753
NLR change rate (range)	0.78	(0.26–22.20)	1.14	(0.21–7.31)	0.460
CRP/Alb change rate (range)	0.74	(0.00–14.11)	2.29	(0.05–34.16)	<0.001
PNI change rate (range)	0.92	(0.21–1.27)	0.84	(0.58–1.40)	0.022

PMI, psoas muscle mass index; nab-PTX, nab-paclitaxel; CA19-9, carbohydrate antigen 19-9; PLR, platelet-to-lymphocyte ratio; NLR, neutrophil-to-lymphocyte ratio; CRP, C-reactive protein; Alb, albumin; PNI, prognostic nutritional index. The continuous variables are expressed as medians and ranges, and the categorical variables are expressed as numbers and percentages.

## Data Availability

The data presented in this article is available from the corresponding author upon reasonable request.
